# UDP-Glucose Dehydrogenases: Identification, Expression, and Function Analyses in Upland Cotton (*Gossypium hirsutum*)

**DOI:** 10.3389/fgene.2020.597890

**Published:** 2021-01-11

**Authors:** Tingting Jia, Qun Ge, Shuya Zhang, Zhen Zhang, Aiying Liu, Senmiao Fan, Xiao Jiang, Yulong Feng, Lipeng Zhang, Doudou Niu, Shen Huang, Wankui Gong, Youlu Yuan, Haihong Shang

**Affiliations:** ^1^State Key Laboratory of Cotton Biology, Key Laboratory of Biological and Genetic Breeding of Cotton, The Ministry of Agriculture, Institute of Cotton Research, Chinese Academy of Agricultural Sciences, Anyang, China; ^2^Zhengzhou Research Base, State Key Laboratory of Cotton Biology, Zhengzhou University, Zhengzhou, China; ^3^Zhengzhou University of Light Industry College of Food and Bioengineering, Zhengzhou, China

**Keywords:** cotton fiber, UDP-glucose dehydrogenase, UDP-glucuronic acid, transgenic *Arabidopsis*, fiber development

## Abstract

UDP-glucose dehydrogenase (UGD; EC1.1.1.22) is a NAD^+^-dependent enzyme that catalyzes the two-fold oxidation of UDP-glucose (UDP-Glc) to produce UDP-glucuronic acid and plays an important role in plant cell wall synthesis. A total of 42 *UGD* genes from four *Gossypium* genomes including *G*. *hirsutum*, *G*. *arboretum*, *G*. *barbadense*, and *G*. *raimondii* were identified and found that the *UGD* gene family has conservative evolution patterns in gene structure and protein domain. The growth of fibers can be effectively promoted after adding the UDP-Glc to the medium, and the *GhUGD* gene expression enhanced. In addition, the transgenic *Arabidopsis* lines over-expressing *GH_D12G1806* had longer root lengths and higher gene expression level than the wild-type plants of *Columbia-0*. These results indicated that *UGD* may play important roles in cotton fiber development and has a guiding significance for dissecting fiber development mechanism.

## Introduction

Cotton fiber is the most important textile raw material. Its primary cell wall components, including pectin, hemicellulose, and cellulose, are mainly derived from a common biochemical precursor, UDP-glucuronic acid (UDP-GlcA; Hofte and Voxeur, 2017), which is responsible for the derivation of about 50% of the cell wall biomass (Zablackis et al., 1995). Plants have evolved two independent pathways for the synthesis of UDP-GlcA. One pathway consists of conversion of UDP-glucose (UDP-Glc) to UDP-GalA catalyzed by UDP-glucose dehydrogenase (UGD, which belongs to the family of NAD^+^-linked oxidoreductase; EC 1.1.1.22; Tenhaken and Thulke, 1996). The other is the more complex myo-inositol pathway, in which cleavage of inositol into D-GlcA catalyzed by myoinositol oxygenase (MIOX; EC 1.13.99.1) is involved. Then, D-GlcA is activated to UDP-GlcA (Loewus et al., 1962; Seitz et al., 2000; Ute et al., 2004; Kanter et al., 2005; Pieslinger et al., 2010). Studies ever demonstrated that down-regulation of *MIOX* genes in the *miox1/2/4/5*-mutants has no effect on cell wall composition in *Arabidopsis thaliana*. Further analysis revealed that an alternative pathway to UDP-GlcA via UDP-Glc is compensatorily up-regulated in these mutants (Endres and Tenhaken, 2011).

The enzyme UGD is a key factor in converting UDP-Glc to UDP-GlcA (Tenhaken and Thulke, 1996). It catalyzes irreversible loss of 4-electrons in UDP-Glc and finally oxidizes it to UDP-GlcA, with a concomitant production of two NADH molecules from NAD^+^. UDP-GlcA is subsequently converted to UDP-D-xylose (UDP-Xyl), of which the corresponding sugar moieties provide abundant components for the synthesis of pectins and hemicelluloses (Reiter, 2008). *UGD* was cloned in *eukaryotes* for the first time (Campbell et al., 1997) and subsequently cloned in higher plants such as soybean (Tenhaken and Thulke, 1996), *A. thaliana* (Klinghammer and Tenhaken, 2007), tobacco (Bindschedler et al., 2005), and poplar (Johansson et al., 2002). *UGDs* have different locations and expression levels in different species and tissues. In soybean, the *UGDs* have high expression level in radicle, while low in epicotyl and spire. The expression pattern of the *UGDs* in different developmental stages indicated that UGD is a key regulator for the availability of hemicellulose precursors (Tenhaken and Thulke, 1996). Analyses of four *UGD* genes in *A. thaliana* revealed that *AtUGD1* is a pseudogene, and *AtUGD2* has the highest expression level in cotyledons and hypocotyls but less in root and *AtUGD3* has the highest enzyme activity (Klinghammer and Tenhaken, 2007). *UGD* plays a key role in the expression process from UDP-glucose to UDP-Xyl in tobacco (Bindschedler et al., 2005). The different expression level of *UGDs* in different parts of poplar development indicated that it played a key role in the spire and immature xylem development (Johansson et al., 2002).

Although a few functional studies of *UGD* genes were performed in some plant species, *UGD* behavior and function in *Gossypium* species is still poorly understood. In this study, a series of bioinformatics analyses including physicochemical properties, evolutionary relationships and gene structure, conserved motifs, and *UGD* domain features were performed. Expression analyses of *UGD* genes, including biochemical experiments, subcellular localization, and functional verification of transgenic *A. thaliana*, were also performed. These results indicated that *UGDs* play an important role in the conversion of UDP-Glc to UDP-GlcA and cotton fiber development, which provides fundamental basis for fiber quality improvement.

## Materials and Methods

### Plant Materials

The plants of upland cotton cultivars 0-153, an upland cotton germplasm line with high-quality fibers, and sGK9708, an upland cotton cultivar with low-quality fibers, were grown under experimental field conditions in Zhengzhou, China (Zhang Z. et al., 2020). The flowers were tagged on the day of anthesis as 0 day post anthesis (DPA) flowers. Developing bolls from the tagged flowers at 5, 10, 15, and 20 DPA were harvested and peeled out with a sterile scalpel to get fiber samples. Fiber samples were immediately dipped into liquid nitrogen and then stored at −80°C conditions for subsequent real-time quantitative PCR (qRT-PCR) assays. Plants of *Nicotiana benthamiana* were grown in the greenhouse with a 16/8 h light/dark cycle at 22°C, 60% relative humidity, and light intensity of 100–120 μmol m^–2^ S^–1^.

### Transcriptomic Data Analysis

The transcriptome data of TM-1, a standard genetic line of upland cotton, was downloaded from NCBI SPA database (accession number PRJNA490626, https://www.ncbi.nlm.nih.gov/bioproject/PRJNA490626; Hu et al., 2019). RNA-Seq raw data of sGK9708 and 0-153 derived from Zhang Z. et al. (2020) were reanalyzed as described previously by Zou et al. (2019). The mapping value of fragments per kilobase of exon per million reads (FPKM) was calculated using the Cufflinks program. Gene expression levels were analyzed by the log_2_ (FPKM + 1) method. FPKM value greater than five was used as a threshold to declare a gene expression. Gene expression patterns were visualized with heat maps prepared using the R (3.3.0) software package^1^.

### RNA Isolation and cDNA Synthesis

Total RNA samples were extracted as described in the kit instructions of the RNAprep Pure Plant Kit (TIANGEN, Beijing, China) and treated with RNase-free DNase I. RNA concentration was calibrated using NanoDrop 2000 (Thermo Scientific), and RNA quality was monitored by agarose gel electrophoresis. The absorbance ratio of A260/280 was used as an indicator of protein contamination. The RNA samples that ultimately meet the minimum eligibility criteria were applied to reverse transcription. First cDNA strand was synthesized using the TransScript All-in-One First-Strand cDNA Synthesis SuperMix for qPCR (Trans, Beijing, China). The concentration of cDNA was calibrated to 100 ng/μl for qRT-PCR experiments.

### The Real-Time Quantitative PCR Analysis

Quantitative real-time PCR analysis of candidate gene expression in cotton tissues was performed using SYBR Green (Roche, 9115 Hague Road, Indianapolis, United States) on a LightCycler^®^ 480 System (Roche). Upland cotton *GhHistone3* (*AF024716*) was used as an internal reference gene (Xu et al., 2004). Primers for candidate genes and internal references are detailed in Supplementary Table 3. Each reaction was carried out in a 384-well plate in a volume of 10 μl. The amplification was performed in a two-step cyclic reaction process as described by Ren et al. (2017). Relative expression levels were analyzed using the LightCycler^®^ 480 Gene Scanning Software. Gene expression levels were calculated according to the 2^–ΔΔCt^ method based on three independent PCR amplifications (Livak and Schmittgen, 2001).

### Ovule Culture and Chemical Treatment

Ovule culture was performed using ovules from sGK9708. Cotton flowers were tagged on the day of anthesis as 0 DPA flowers, and ovules were sampled from the developing bolls from the tagged flowers at 1 DPA. After harvest, ovules were immediately sterilized and cultured in BT medium (Beasley, 1973) that contains 0 (as control), 0.5, 1, and 5 μM nucleotide sugar UDP-Glc, respectively. The culture was incubated at 30°C in the dark. The nucleotide sugar UDP-Glc (CAS: 28053-08-9) was purchased from Sigma-Aldrich (Sigma Corporation of America). UDP-Glc was prepared with double-distilled water (ddH_2_O) to a stock concentration of 5 mM and sterilized by filtration through a 0.22-μm Millex filter (Millipore). In the medium preparation, the stock solution was diluted to the required working concentration corresponding to the treatments. At 7 DPA, the ovules in culture were sampled to evaluate the phenotypes of fiber development. Because the fibers are too fragile to stand any mechanical processing in the early stage of culture, the phenotypes of fiber development were measured intact using digital images in the form of fiber mass areas to elucidate the effect of precursor substances on fiber development. Digital images of the fibers intact on the cultured ovules were recorded with Leica DFC7000 T stereo microscope (Leica M165 FC) under the same parameters of shooting distance, light source, resolution, and magnification. The area of the fiber mass was measured and calculated using ImageJ software (version 1.52v). All experiments were in three independent replications, and 30 ovules per row were selected for fiber mass area assessment.

### Genetic Sequence Retrieval and Phylogenetic Analysis

To identify candidate gene family members, the hidden Markov model (Pfam: PF00984\PF03720\PF03721) was downloaded from the Pfam website and renamed as UGD.hmm. The gene sequence retrieval was performed with the UGD.hmm model using the hmmer search program in HMMER 3.0 software (Finn et al., 2011). The UGD gene family sequences of *A. thaliana* were used to retrieve the genome data of nine species. The preliminary identification of UGD protein ID was searched in the following published protein sequences: *Gossypium hirsutum* (Hu et al., 2019)^2^, *Gossypium arboretum* (Du et al., 2018)^3^, *Gossypium barbadense* (Hu et al., 2019)^4^, and *Gossypium raimondii* (Paterson et al., 2012)^5^. The genomes for comparison analysis, including *Theobroma cacao*, *Oryza sativa*, *A. thaliana*, *Populus trichocarpa*, and *Brachypodium distachyon* genomes were downloaded from ePhytozome 2.1 database^6^. The Basic Local Alignment Search Tool (BLAST) was performed to search the protein sequences from the four species using protein–protein BLAST (BLASTP)^7^ program. The results obtained were compared to the results of the previous hmmer search. Since the genes in the annotation files were corresponded to multiple transcripts, redundant transcripts were manually discarded.

### Phylogenetic Tree Construction, Motif, and Exon/Intron Structure Analyses

Phylogenetic analysis was performed using the conservative regions that were obtained through multiple sequence alignments. The phylogenetic tree was constructed using the MEGA 7 program (Tamura et al., 2013) using the neighbor-joining (NJ) method with 1,000 lead replicates.

Structural information of the *UGD* genes was obtained from the GFF3 file, and the predicted coding sequence was aligned with the corresponding genomic sequence using the Gene Structure Display Server (GSDS; Hu et al., 2015) to search for the exon/intron structure. The conservative motifs of candidate genes were determined using the online program MEME^8^, and the identified motifs were annotated using the InterProScan program (Hu et al., 2015). TBtools^9^ was used to visualize gene motifs. The isoelectric point (PI) and molecular weight (Mw) were calculated online the ExPASy website^10^.

The values of non-synonymous substitution rate (Ka) to synonymous substitution rate (Ks) were evaluated using KaKs Calculator 2.0 software to analyze the selection pressure of UGD genes.

### Promoter Region Retrieval and Analyses

Promoter regions of about 2,000 bp upstream of the start codon of the candidate gene were extracted from the genomic sequence of *G. hirsutum*, and the *cis*-acting regulatory elements (CARE) were thus analyzed using the online PlantCARE software^11^ (Lescot et al., 2002). In addition, all CAREs in the promoter region of the gene of interest were classified according to their efficacies in plant responses and development mechanisms.

### Subcellular Localization Assay

The coding region of *GH_D12G1806* was isolated from the cDNA template, which was derived from the mixture of fiber samples of 5DPA, 10DPA, 15DPA, and 20DPA developing bolls of sGK9708, by hi-fi PCR amplification using primer pair of F: 5′-TCTAGAATGGTGAAGATCTGTTGCATCG-3′ and R: 5′-TTAATTAATGCCACAGCTGGCATGTCC-3′. The amplified product was cloned into the GFP-containing vector *pCambia2300* in an expression cassette driven by the 35S promoter according to Witte et al. (2004). Tobacco (*N. benthamiana*) leaves were co-infiltrated with *Agrobacterium* strains containing the GFP coupled with *pCambia2300* according to Witte et al. (2004). After 48 h of infection following the procedure described previously (Maiti et al., 1988; Goodin et al., 2002), leaves were observed using an Olympus FV1200 (Olympus, Tokyo, Japan) confocal laser scanning microscope. Fluorescent images of EGFP were collected to detect the subcellular localization of *UGD*.

### Transformation of *A. thaliana*

*GH_D12G1806* was isolated using the same cDNA template and procedures as described in the *Subcellular Localization Assay* section with primer pairs of forward: 5′-TCTAGAGGTGAAGATCTGTTGCATTGGA-3′ and reverse: 5′-GAGCTCTGCAGGCATGTCCTTGAGC-3′. The amplified product was cloned into vector *pCambia2300* in an expression cassette driven by the 35S promoter. Then, the constructed vector was introduced into *Agrobacterium tumefaciens GV3101* (Clough and Bent, 1998). Floral dip method was used for *Agrobacterium*-mediated transformation into *A*. *thaliana*. Phenotypic identifications were performed in T_3_ generation plants of transgenic *A*. *thaliana*.

## Results

### Identification of the *UGD* Gene Family Numbers

A total of 42 total *UGD* genes from *G. hirsutum*, *G. arboreum*, *G. barbadense*, and *G. raimondii* genomes were identified, including 15 *GhUGDs*, 15 *GbUGDs*, 6 *GaUGDs*, and 6 *GrUGDs* (Supplementary Table 1). In order to elucidate their evolutionary and phylogenetic relationships, *UGD* genes from extra five species, including four in *A. thaliana*, four in *T. cacao*, four in *P. trichocarpa*, two in *B. distachyon*, and five in *O. sativa*, were identified and analyzed (Supplementary Table 2).

### Phylogenetic Analysis, Gene Structure, and Protein Domain of the *UGD* Gene Family

**FIGURE 1:**
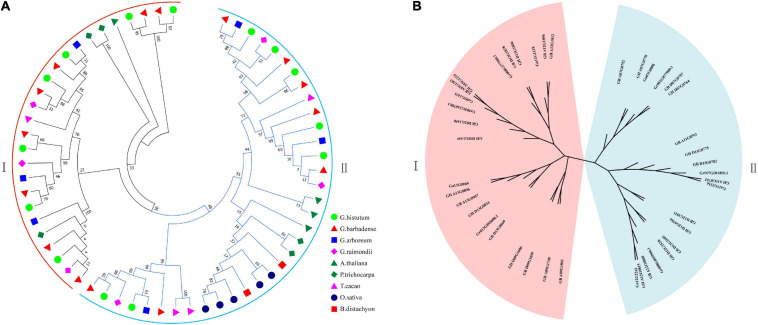
Phylogenetic trees of *UGDs*. **(A)** Phylogenetic tree of 61 *UGDs* from 9 species, including *G. hirsutum*, *G. barbadense*, *G. arboreum*, *G. raimondii*, *A. thaliana*, *T. cacao*, *P. trichocarpa*, *B. distachyon*, and *O. sativa*; **(B)** phylogenetic tree of 42*UGDs* from four *Gossypium* species.

**FIGURE 2:**
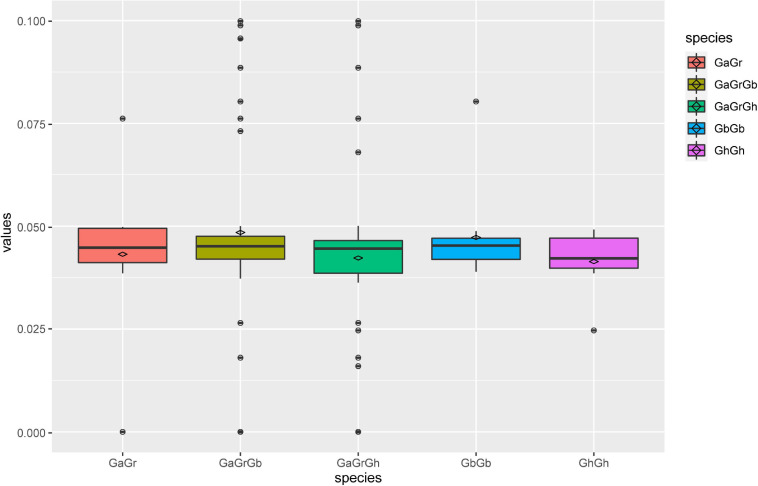
Multiple comparison of Ka/Ks values of homologous pairs of UGD family in four *Gossypium* species.

According to the topological structure of the phylogenetic tree, the *UGD* genes in the nine species can be assorted into two subfamilies, *UGD*-I and *UGD*-II, containing 28 and 33 members, respectively, (Figure 1A). The result of phylogenetic analysis indicates that the *UGDs* have a closer evolutionary relationship among the four *Gossypium* species as compared with those among other species. Further phylogenetic analysis of *UGDs* in the four cotton species indicates that the *UGD*-I are assorted into four subgroups, while the *UGD*-II three (Figure 1B). Each subgroup of *Gossypium UGDs* consists of six members including one from A genome (*G. arboreum*; Du et al., 2018; see text footnote 3), one from D genome (*G. raimondii*; Paterson et al., 2012; see text footnote 5), two from *G. hirsutum*, and two from *G. barbadense*. As both *G. hirsutum* and *G. barbadense* are consisted of A_t_ and D_t_ subgenomes, each subgenome provides one member in a sub-group of *UGD* family. There is one subgroup in *UGD*-I that has four *UGDs*, in which there is no *UGD* from *G. arboreum* and *G. raimondii* identified. The *UGD*-II has one subgroup that has eight *UGDs* including three from *G. hirsutum* and three from *G. barbadense* (Figure 1B). The PI values and Mws of the four cotton *UGDs* range from 5.00 to 7.39 and from 29.41 to 56.50 kD, respectively. CDS lengths of most *UGD* genes are between 777 and 2,544 bp. The protein sequences that these genes encode consist of 258–847 amino acids (aa; Supplementary Table 1). The CDS and protein lengths of the *UGD* genes suggest that the *UGD* genes are in a conservative mode in genome evolution among the four *Gossypium* species. Ka/Ks value analysis reveals that the Ka/Ks values of most UGD homologous pairs are below 0.1 (Figure 2), indicating purifying selection pressure of UGD gene family during its evolution (Ge et al., 2020).

**FIGURE 3:**
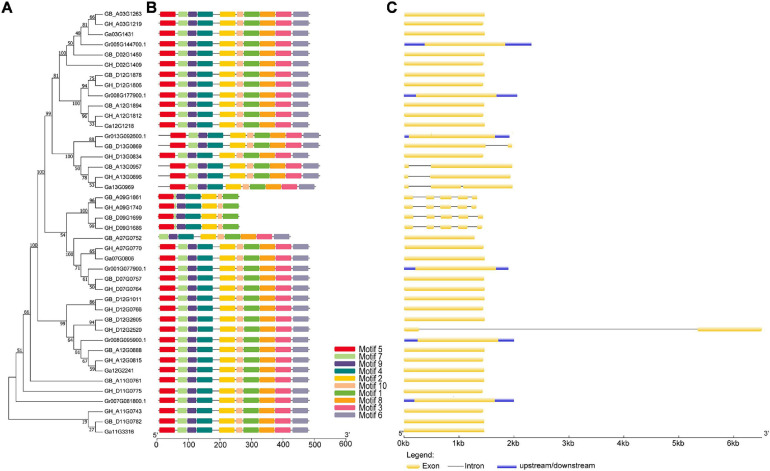
Evolutionary relationship, motif, and gene structure analysis of the UGD family in *Gossypium* species. **(A)** Phylogenetic relationships; **(B)** conserved motifs of UGD proteins. Motif compositions were determined using MEME. **(C)** Gene structure of UGD genes. The neighbor-joining tree was constructed with MEGA 7. I and II indicate the two subfamilies of UGDs. Yellow and blue bars represent CDS and UTRs, respectively.

In order to comprehensively study the phylogenetic relationship among *UGD* genes in four cotton species, we performed analyses of gene structures and protein domains. The results reveal that *UGD* genes share similar motif and genetic structures (Figures 3B,C). It demonstrates that the exon number variations are exclusively identified in *UGD*-I subfamily, in which its members contain one–five exons, and most genes only have only one exon. UTR structures are identified exclusively in *UGD* genes of *G. raimondii*. These results suggest that *UGD* family in *Gossypium* is evolutionarily conservative, while some *UGD* genes in *G. raimondii* may be evolutionarily active (Figure 3C).

### *Cis*-Acting Regulatory Elements in the Promoter Regions of *UGD* Genes

**FIGURE 4:**
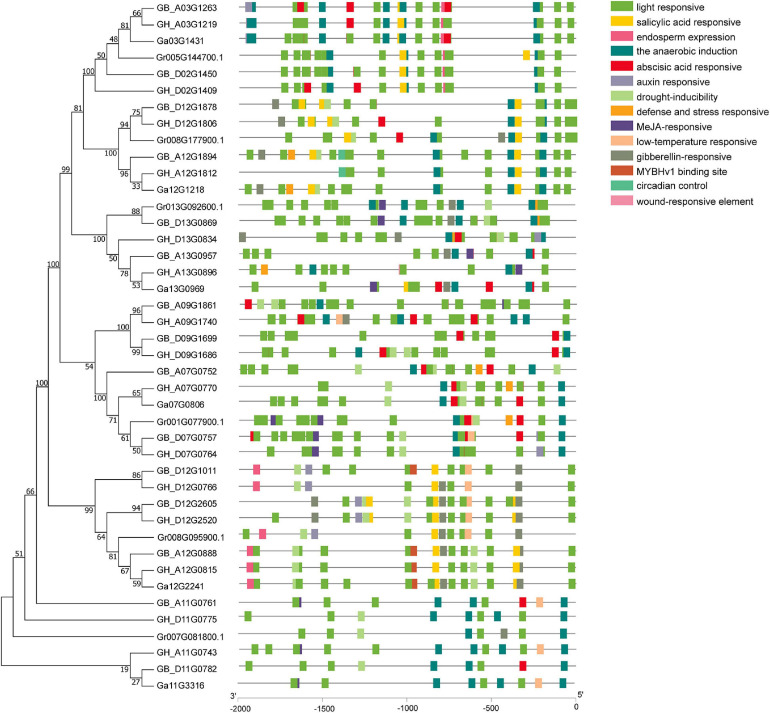
*Cis*-acting regulatory elements analysis of cotton *UGD* genes.

Analysis of the CAREs within the 2,000 bp upstream region from the start codon (ATG) of *GhUGDs* revealed that each gene promoter region contains multiple CAREs. Totally, 14 potential CAREs are identified in *UGD* promoter regions. The composition and distribution of CAREs vary significantly across the whole UGD gene family. The homologous *UGD* genes have same or similar CAREs composition and distribution in the same subfamily. Detailed analyses reveal these CAREs mainly consist of light response, hormone response, and abiotic stress responses (including drought, low temperature, defense, and stress) and specific organ/tissue development elements (Figure 4). Light-responsive elements include AT1-motif, TCT-motif, GT1-motif, AAAC-motif, TCCC-motif, GATA-motif, GA-motif, GA-motif, TGA-element, I-box, MRE, Box4, G-Box, ACE, chs-CMA, and Gap-box. Hormone-responsive elements include salicylic acid-responsive element (TCA-element), gibberellin-responsive elements (including P-box, GARE-motif, and TATC-box), abscisic acid-responsive element (ABRE), and auxin-responsive elements (AuxRR-core, TGA-element). Stress response elements include TC-rich repeats (as defense and stress responsive elements), MBS (drought-inducibility elements), and LTR (low-temperature-responsive element). Overall, these results suggest that the distribution and composition of these diverse CAREs may shape the expression of *UGD* family genes in responses to various stimuli of light, hormones, abiotic stresses, defense signal transduction, and cotton plant development.

### Expression Pattern Analyses of *UGD* Genes in Developing Fibers

**FIGURE 5:**
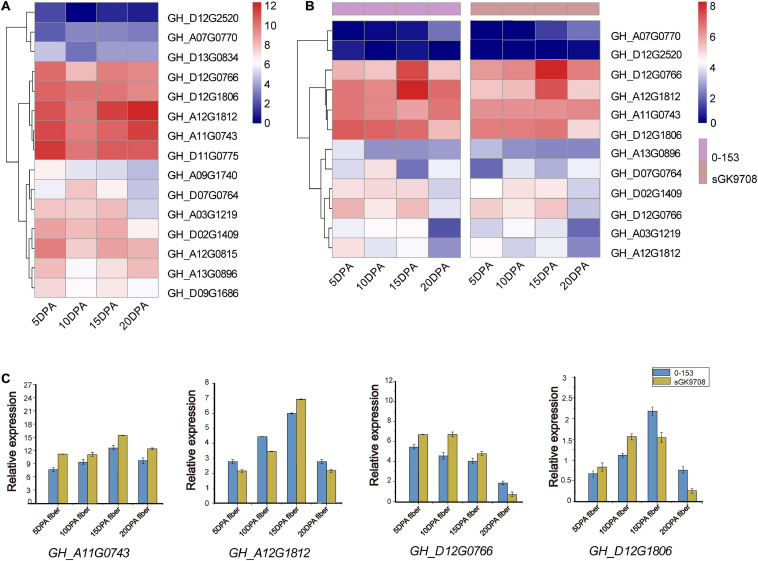
Gene expression patterns among different varieties. Heat maps of gene expression levels in cotton fibers are shown for fibers (indicated at the bottom of each lane). **(A)** Heat map of *UGD* gene expression levels in fiber samples collected on 5, 10, 15, and 20 DPAs in TM-1 (Hu et al., 2019). The expression level was scaled using the log_2_ (FPKM + 1) method. **(B)** Heat map of *UGD* gene expression levels in fiber samples collected on 5, 10, 15, and 20 DPAs in two upland cotton varieties 0-153 and sGK9708. **(C)** qRT-PCR analysis of some *UGD* members in 0-153 and sGK9708. Expression levels are shown relative to the reference gene *GhHis3.* Error bars represent the standard deviations of three independent experiments.

To explore the potential function of *UGD* genes in developing fibers of upland cotton, we analyzed the gene expression patterns of the *UGD* gene families using two cotton fiber transcriptome datasets. One is dataset of *TM-1* from NCBI SPA database (accession number PRJNA490626, https://www.ncbi.nlm.nih.gov/bioproject/PRJNA490626; Hu et al., 2019) and the other is RNA-Seq raw dataset of sGK9708 and 0-153 derived from Zhang Z. et al. (2020). The results of expression analysis show that *GH_D12G0766*, *GH_D12G1806*, *GH_A12G1812*, and *GH_A11G0743* from *G. hirsutum* have significant FPKM values in developing fibers both in *TM-1* (Figure 5A and Supplementary Table 3) and in current commercial cultivars sGK9708 and 0-153 (Figure 5B and Supplementary Table 3). The result indicated that these genes had a similar expression profiles across various materials in our study. qRT-PCR verifications further confirmed their expression specificities in developing fibers of sGK9708 and 0-153 (Figure 5C and Supplementary Table 4) and their expression consistencies with transcriptome analysis. Promoter response elements affect downstream gene expression (Li and Zhang, 2014). In these genes, by observing the promoter elements (Figure 4), it was found that only the *GH_D12G1806* promoter region contained abscisic acid (ABA) response elements. Studies have shown that shoot ABA application promotes root growth by accelerating both cell division and elongation of the root tips (Lin and Kao, 2001). ABA synthesis regulates fiber development (Zhang G. et al., 2020). Therefore, we chose *GH_D12G1806* as our candidate genes for the next step of functional verification.

### Effect of UDP-Glc on Fiber Development

**FIGURE 6:**
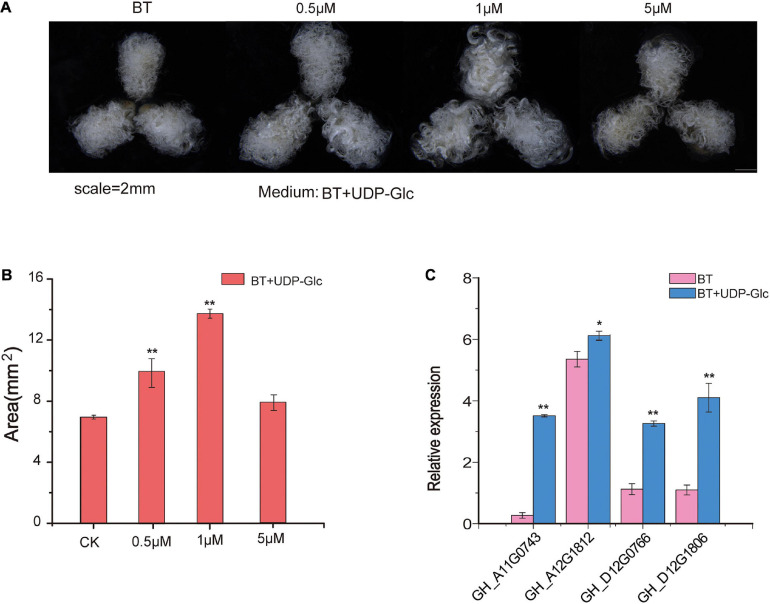
Phenotypic evaluation of fibers after addition of UDP-Glc precursors to ovule culture. **(A)** Phenotypic variations of the area of fiber mass in different concentrations of UDP-Glc precursors applied in the culture media. **(B)** Statistical analysis of the phenotypic variations of the area of the fiber mass in different concentrations. **P < 0.01 **(C)** The qRT-PCR verification of relative expression levels of *GH_D12G1806* ovules at 7 DPA after UDP-Glc treatments. CK represents control in which UDP-Glc is not applied and ovules only grows on BT medium. *P < 0.05, **P < 0.01.

UDP-glucose is the substrate of *UGD* to synthesize UDP-GlcA, which is a precursor of pectin (Hofte and Voxeur, 2017). To monitor the effects of UDP-Glc on fiber development, different concentrations of UDP-Glc were added to the media of ovule culture. The areas of fiber cluster under ovule culture were evaluated, and the result shows that the effects of UDP-Glc are significantly different at different concentrations at 7 DPA after ovule culture as compared to that of controls (Figures 6A,B). It shows that 1 μM of UDP-Glc has the most significant effect on fiber development. The growth of fibers can be effectively promoted in the ovules that are cultured on the medium containing UDP-Glc. qRT-PCR analysis demonstrates that the expression patterns of *GhUGD* genes are significantly up-regulated in UDP-Glc treatment as compared to those in control, which shows a consistency with our speculations (Figure 6C).

### Subcellular Localization of the Expression Product of *GH_D12G1806*

**FIGURE 7:**
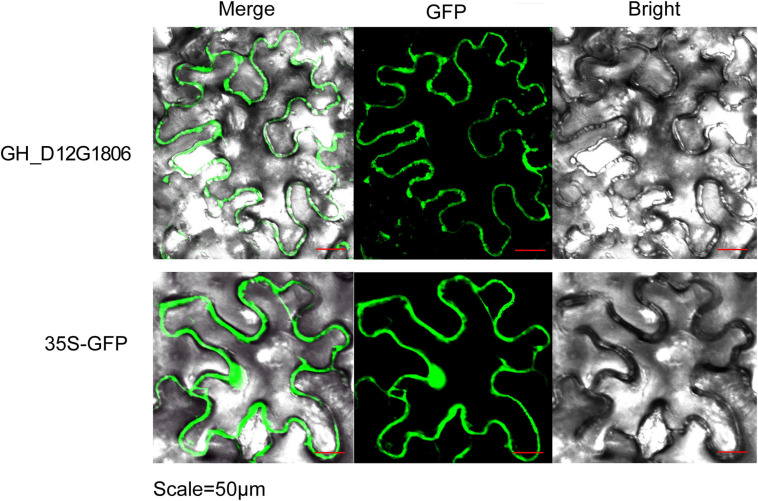
Subcellular localization of *GH_D12G1806* in bright field, fluorescence (GFP), and merged images of tobacco leaves infiltrated with *Agrobacterium*.

To elucidate the subcellular localization of *GhUGDs*, the coding region of *GH_D12G1806* was isolated and constructed into a co-expression construct of *GH_D12G1806*-*GFP*. Leaves of *N. benthamiana* were infiltrated with *A. tumefaciens* transformed with the co-expression construct to detect its transient expression. Confocal imaging of the permeated tobacco leaves shows that the *GhUGD*-GFP signal are present on the cell membrane, indicating that the *UGD* genes are expressed on the cell membrane (Figure 7).

### Over-Expression of *GH_D12G1806* in *A. thaliana*

**FIGURE 8:**
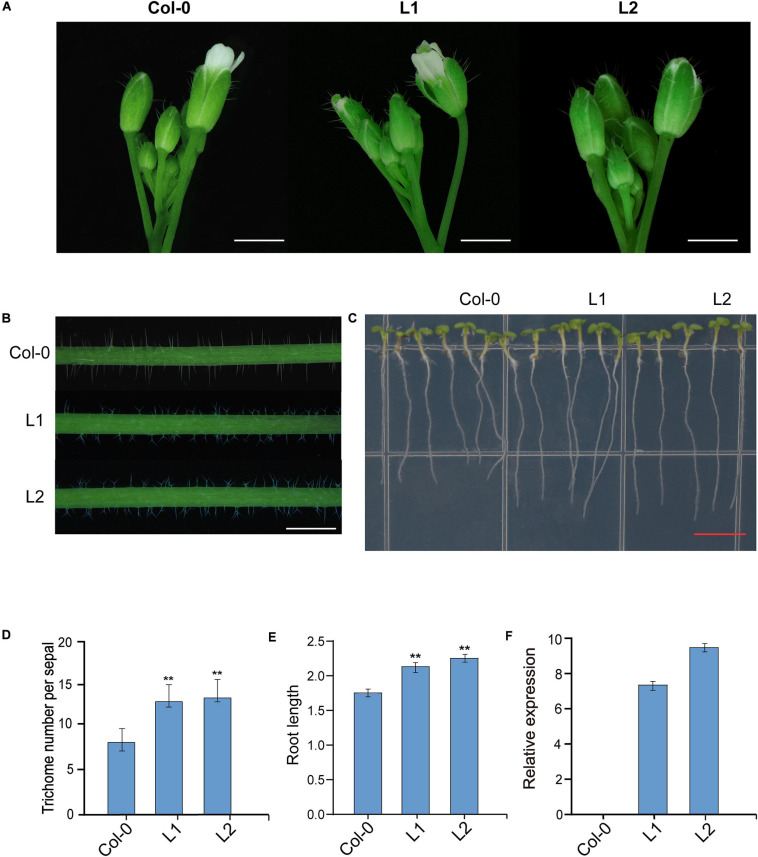
Overexpression of the *GH_D12G1806* gene in *A. thaliana*. **(A)** Trichome initiation on the sepals of wild-type *Col-0* and transgenic lines L1 and L2. Scale bars = 0.1 mm. **(B)** Trichomes on the main inflorescence stems of *Col-0*, L1, and L2 plants. Scale bars = 1.0 mm. **(C)** Root phenotypes of *Col-0*, L1, and L2. Scale bars = 1 cm. **(D)** Statistical analysis of the phenotypic variations of the densities of trichomes in the sepals from *Col-0* and L1 and L2. Error bars represent the SD of three biological replicates. **P < 0.01. Scale bars = 2.0 mm. **(E)** Statistical analysis of the phenotypic variations of root length. Error bars represent the SD of three biological replicates (*n* = 30 each) **P < 0.01. **(F)** qRT-PCR verification of the relative expression of *GH_D12G1806* in *Col-0*, T1, and T2.

To verify *GhUGD* genes function in *A. thaliana*, the coding region of *GH_D12G1806* was constructed in an over-expression cassette driven by 35S promoter. When the over-expression cassette was introduced into *A. thaliana* (*Columbio-0, Col-0*), totally 10 tranformants were obtained in T_0_ generation. Genetic screening in T_2_ generation showed seven single-copy insertion lines were obtained. In T_3_ generation, the phenotypic variations of two transgenic lines L1 and L2 were evaluated (Figure 8). At flowering stage, the number of trichomes on the sepals of transgenic lines L1 and L2 increased significantly, which were 155% and 160% of that of the control plants, respectively, (Figures 8A,D). Compared with the *Col-0*, most of the trichomes on the main stem of L1 and L2 showed bifurcated phenotypes (Figure 8B). The results show that root lengths of L1 and L2 increase significantly by 121% and 125%, respectively, as compared with those of the control *Col-0* plant at 7 days after germination (Figures 8C,E). To confirm these phenotypic variations, the expressions of *GH_D12G1806* in both the transgenic lines (L1 and L2) and wild-type *Arabidopsis* plants were verified through qRT-PCR. The results of qRT-PCR verification demonstrate that there is no detectable expression of *GH_D12G1806* in the control plant and that expression levels in both L1 and L2 are significantly high (Figure 8F). These results indicate that over-expression of *GH_D12G1806* in transgenic lines may promote trichome initiation and root length in the transgenic *Arabidopsis* plant.

## Discussion

UDP-glucose dehydrogenase catalyzes the NAD^+^-dependent two-fold oxidation of UDP-Glc to UDP-GlcA, which is a cell wall-specific biochemical precursor required for the synthesis of much of the cell wall biomass including hemicellulose and pectin (Endres and Tenhaken, 2011), especially in newly formed plant cell walls (Tenhaken and Thulke, 1996; Johansson et al., 2002; Griffith et al., 2004). The *UGDs* had been extensively studied in pathogenic microbes where it played multiple functions including keeping cell wall integrity of *C. neoformans* (Bar-Peled et al., 2004) and maintain virulence of *Cryptococcus neoformans*. In maize, UGDs play an important role in cell wall pentose biosynthesis. However, the characterization and functional analysis of the UGD family has not been performed in *G. hirsutum*.

In this work, *UGD* gene family kept a certain stability in different diploid species including *A. thaliana* (in which four *UGD* genes were identified), *T. cacao* (four *UGD* genes), and *G. arboretum* (six *UGD* genes), and *G. raimondii* (six *UGD* genes; Figure 1A). We also simultaneously identified *UGD* genes in four representative cotton species, two tetraploids of *G. hirsutum* and *G. barbadense* and two diploids of *G. arboreum* and *G. raimondii*, from the ancestors of which the tetraploids were formed 1.5 million years ago (Hu et al., 2019). We identified 15 *UGD* genes each in *G. hirsutum* and *G. barbadense* and 6 *UGD* genes each in *G. arboreum* and *G. raimondii* (Figure 1B). These results indicate that UGD gene family in *G. hirsutum* and *G. barbadense* may expand through duplications during their evolutions, which is a common phenomenon in plant genome evolutions (Schaper and Anisimova, 2015; Ge et al., 2020). On the other hand, gene losses in both allotetraploid diploid species are also observed (Li et al., 2015; Hu et al., 2019). However, how these forces drive the formation of *UGD* gene family is still open to elucidation.

The protein sequences of UGDs are highly conservative in the plant, which can be inferred from the observations that more than 90% sequences were identical between different isoforms (Klinghammer and Tenhaken, 2007). In the current study, bioinformatics analysis of the *GhUGD* gene family revealed conserved motifs and gene structures (Figure 3). Together with purifying selection pressure of UGD homologous pairs, they further illustrate that the *GhUGD* gene family is conservative in evolution. Promoter region analysis revealed that ABRE, a *cis*-acting regulatory element of ABA-promoting ethylene synthesis, was identified in some *GhUGDs*. Ethylene participates in the regulation of specific types of cell growth and promotes elongation of fibroblasts *in vitro* (Shi et al., 2006; Pang et al., 2010). In *A. thaliana*, most genes containing ABRE elements are highly expressed in leaves and roots (Kanter et al., 2005). In the current study, the genes specifically expressed in developing fibers also harbored ABRE in their promoter regions. Therefore, the ABRE effects in *GhUGD* expression in developing fibers are worth of further studies.

UDP-GlcA can be synthesized alternatively by the MIOX or UGD in *A. thaliana* (Seitz et al., 2000; Kanter et al., 2005). However, biochemical labeling experiments of cell cultures with *A. Thaliana* suggested a dominance of the UGD pathway for UDP-GlcA formation (Sharples and Fry, 2007). Down-regulation of UDP-GlcA biosynthesis leads to swollen plant cell walls and severe developmental defects associated with changes in pectic polysaccharides (Reboul et al., 2011). Also, recent research has demonstrated that the double mutant *ugd2ugd3* shows defects in seedling development, slow growth, dwarfism, and low seed-set rate, suggesting that UGD enzymes are of great importance for the integrity of the cell wall. Although *UGD* genes are mostly expressed in roots and leaves in *A. thaliana*, the results of current study revealed *UGD* genes that are also highly expressed in cotton fiber (Figure 5). The potential functions of these genes are still open to further analysis and verifications. Some studies suggested that on genome-wide scale, homoeologous gene pairs may not show expression bias (Zhang et al., 2015); however, in a specific tissue or developmental stage, the biased expression might be correlated to subfunctionalization of these homoeologous genes (Zhang et al., 2015). These expression profiling might indicate the functional relevance. A previous study indicated that the exogenous application of UDP-Glc into ovule culture solution significantly promoted cotton fiber elongation and increased *UGD* gene expression (Pang et al., 2010). The transcriptome analysis in the current study at least identified four *GhUGD* genes highly expressed in developing fibers. Subcellular localization of *GH_D12G1806* from *G. hirsutum* revealed that its functional site was mainly located in the cell membrane, which inferred that the enzyme UGD may play an important role in cotton fiber development. When UDP-Glc was applied to the ovule culture solution, fiber mass growth was promoted in the culture with UDP-Glc and *UGD* gene expressions increased (Figure 6), which is consistent with the results of Pang et al. (2010). The results also suggest that different UDP-Glc concentrations may modulate its final effect on the development of fibers.

Plant growth is largely determined by cell elongation, a process that is strongly controlled at the level of the cell wall. Down-regulation of UGD-GlcA reduces pentose contents in the cell walls (Klinghammer and Tenhaken, 2007), which suggests that UGD as a key enzyme for the synthesis of UDP-GlcA may play an important role in cell wall synthesis. Examination of UGD function in *A. thaliana* revealed that the antisense *GbUGD6* lines had shorter roots, deferred blossoming, and activities of associated enzymes were also affected by UGD reduction, compared to wild-type plants (Han et al., 2016). In this study, we transformed *GH_D12G1806* into *A. thaliana* (*Columbia-0, Col-0*), and the over-expression transgenic lines L1 and L2 had longer root and more trichomes on sepals of flowers than control plant of *Col-0* (Figures 8A–E), indicating that the promoted expression of *GH_D12G1806* may enhance root cell growth and trichome initiation. However, what the mechanism is between *GH_D12G1806* expression and cotton fiber development is still unclear. As trichomes and fibers are all single epidermic cells, they share similar initiation and growth mechanisms (Wang et al., 2019). Possibly, *GH_D12G1806* works the same way it does on trichomes.

## Conclusion

In this study, a total of 42 *UGD* genes based on the genome information of *G. raimondii*, *G. arboreum*, *G. hirsutum*, and *G. barbadense* were identified. The family could be divided into two groups based on the phylogenetic tree and gene structures. UGD gene family in *G. hirsutum* and *G. barbadense* may expand through duplications during their evolutions, which is a common phenomenon in plant genome evolutions. When UDP-Glc was applied to the ovule culture solution, fiber mass growth was promoted in the culture with UDP-Glc and UGD gene expressions increased. The over-expression transgenic lines L1 and L2 had longer root and more trichomes on sepals of flowers than control plant of *Col-0*, indicating that the promoted expression of *GH_D12G1806* may enhance root cell growth and trichome initiation. These results indicate that UGD may play an important role in cell wall synthesis and its mechanism on cotton fiber development and fiber quality formation is worth of further dissecting.

## Data Availability Statement

The raw data supporting the conclusions of this article will be made available by the authors, without undue reservation.

## Author Contributions

HS and YY designed the experiments and provided the resources for all experiments. TJ coordinated the experiments. TJ, QG, SH, and WG analyzed the data and wrote the manuscript. SF and QG contributed in the plant material preparation and RNA extraction. SZ and XJ contributed to multiple sequences alignments and phylogeny analysis. YF, LZ, and DN contributed to qRT-PCR analysis. ZZ, AL, and WG discussed the results and commented on the manuscript. All authors read, edited, and approved the current version of the manuscript.

## Conflict of Interest

The authors declare that the research was conducted in the absence of any commercial or financial relationships that could be construed as a potential conflict of interest.

## Footnotes

1 https://CRAN.R-project.org/package=pheatmap

2 https://cottonfgd.org/about/download/assembly/genome.Ghir.ZJU.fa.gz

3 https://cottonfgd.org/about/download/assembly/genome.Garb.CRI.fa.gz

4 https://cottonfgd.org/about/download/assembly/genome.Gbar.ZJU.fa.gz

5 https://cottonfgd.org/about/download/assembly/genome.Grai.JGI.fa.gz

6 http://www.phytozome.net

7 https://blast.ncbi.nlm.nih.gov/Blast.cgi

8 http://meme-suite.org/

9 https://github.com/CJ-Chen/TBtools

10 http://web.expasy.org/compute_pi/

11 http://bioinformatics.psb.ugent.be/webtools/plantcare/html/

## Supplementary Material

Supplementary Table 1 Chromosomal locus ID and length of UGD proteins in four cotton species.

Supplementary Table 2 *UGD* gene family in nine species.

Supplementary Table 3 *UGD* gene family and their PFKM values in TM-1, 0-153, and sGK9708.

Supplementary Table 4 Primer pair sequence for qRT-PCR analysis of UGD genes and internal reference *GhHis3* gene.

## Abbreviations

UDP-Glc: UDP- α-D -glucose
UDP-GalA: UDP- α-D -galacturonic acid
ABA: abscisic acid
UGD: UDP-glucose dehydrogenase
MIOX: inositol oxygenase
GlcAK: glucuronokinase
USP: UDP-sugar pyrophosphorylase
DPA: days post anthesis
FPKM: fragments per kilobase of exon per million reads
BLAST: Basic Local Alignment Search Tool
GSDS: Gene Structure Display Server
PI: isoelectric point
Mw: molecular weight
cDNA: complementary DNA.
